# Aging, Obesity, and Inflammatory Age-Related Diseases

**DOI:** 10.3389/fimmu.2017.01745

**Published:** 2017-12-07

**Authors:** Daniela Frasca, Bonnie B. Blomberg, Roberto Paganelli

**Affiliations:** ^1^Department of Microbiology and Immunology, University of Miami Miller School of Medicine, Miami, FL, United States; ^2^Dipartimento di Medicina e Scienze dell’Invecchiamento, Università degli Studi ‘G. d’Annunzio’ Chieti-Pescara, Chieti, Italy

**Keywords:** aging, obesity, inflammation, type-2 diabetes, rheumatoid arthritis

## Abstract

The increase in the prevalence of obesity represents a worldwide phenomenon in all age groups and is pathologically and genetically correlated with several metabolic and cardiovascular diseases, representing the most frequent age-related diseases. Obesity superimposed on aging drastically increases chronic low-grade inflammation (inflammaging), which is an important link between obesity, insulin resistance, and age-associated diseases. Immune cells of both the innate and the adaptive immune systems infiltrate the adipose tissue (AT) and during obesity induce inflammatory responses associated with metabolic switches and changes in phenotypes and function of immune cell subsets. Obesity poses new health problems especially when it occurs in the context of other diseases, many of them frequently affect elderly subjects. An emerging problem is the decreased proportion of patients with obesity achieving clinical response to therapy. In this review, we will discuss the reciprocal influences of immune cell and AT inflammation in aging and age-associated diseases and the complex relationship of nutrient and energy-sensing homeostatic checkpoints, which contribute to shape the phenotype of the AT. We will specifically examine type-2 diabetes, rheumatoid arthritis, osteoarthritis, cognitive impairment, and dementia, where obesity plays a significant role, also in shaping some clinical aspects.

## Introduction

The increase in prevalence of overweight and obesity represents a worldwide phenomenon, which is associated with several chronic diseases such as type-2 diabetes (T2D), cancer, rheumatoid arthritis and osteoarthritis (OA), cognitive impairment and dementia, and those affecting the cardiovascular (CV) system.

The global obesity pandemic affects all age groups. Recent studies examining body mass index (BMI) data in 68 million people in 195 countries showed both increased prevalence and disease burden of high BMI subjects globally over the past 20 years (1). Although the prevalence of obesity among children is lower than in adults, its rate of increase exceeds that of adults (2). The global burden of disease related to high BMI is calculated in individuals without underlying conditions, and it increases at a slower pace in adults mainly because of the reduction of other risk factors for CV diseases and for effective clinical intervention. However, increased BMI has been shown to be pathogenetically related to several diseases. Among these, insulin resistance (IR) and T2D have a strong link to obesity, and the metabolic syndrome represents a cluster of risk factors for severe CV events (coronary artery disease, stroke). Obesity superimposed on aging represents an additional risk factor for older age groups in which the prevalence of chronic disease as well as the occurrence of complications increases (3–5). The disease burden of high BMI in children (≤18 years of age) has not been addressed in the same detail.

The aging process is characterized by a state of chronic inflammation, known as inflammaging. Several factors contribute to inflammaging, including polymorphisms in the promoter regions of pro-inflammatory genes, chronic stimulation of immune cells with viruses such as cytomegalovirus, changes in the gut microbiome, and increased permeability from the intestine [reviewed in Ref. (6)]. It has been recently proposed that continuous engagement of innate receptors by endogeneous signals such as damage-associated molecular patterns drives a chronic state of background inflammation, which needs to be counterbalanced by anti-inflammatory mechanisms. Cellular senescence and the acquisition of the senescence-associated secretory phenotype (SASP) by fibroblasts (7) and endothelial (8) and immune cells (9–11) has also been pinpointed as a significant contributor to inflammaging. Cell senescence induces the accumulation of terminally differentiated B, T, and NK cells with dysregulated function through the activation of pathways integrating senescence and energy-sensing signals.

Inflammaging is an important link among obesity, IR, aging, and age-associated diseases such as cognitive impairment, atherosclerosis, cancer, and autoimmunity. Elevated pro-inflammatory cytokines are associated with decreased insulin sensitivity. Chronic low-grade (sterile) inflammation causes IR, which leads to the transition from metabolically normal obesity to metabolic syndrome. This occurs through both systemic inflammation and metaflammation (12), a process whereby excess nutrients promote chronic low-grade inflammation, and whose metabolic hallmarks are high levels of lipids, free fatty acids (FFAs), glucose, and reactive oxygen species (ROS).

Immune cells of the innate and adaptive immune systems infiltrate insulin responsive tissues, such as the visceral adipose tissue (VAT) and with obesity incite inflammatory responses. Immune cells (macrophages, T, B, NK, NKT cells, and neutrophils) have been implicated in adipose tissue (AT) inflammation and IR (13–17). Inflammation leads to local and systemic increases in pro-inflammatory molecules, such as tumor necrosis factor (TNF)-α, interleukin (IL)-1β, IL-6, interferon (IFN)-γ, inflammatory adipokines, chemokines, and FFAs [reviewed in Ref. (16)].

## Links of Obesity to Insulin Resistance (IR) and T2D

IR is the lack of appropriate response to circulating insulin in several tissues, including liver, muscle, and AT (18). It frequently associates with obesity, hypertension (integrating features of the metabolic syndrome), and CV disease and typically precedes the onset of T2D. In the pancreas, β-cells adapt to hyperglycemia with an expansion of the total β-cell mass and with increased secretion of insulin (hyperinsulinemia), which is able not only to control normal levels of glycemia but also can induce β-cell stress, causing β-cell failure, and then T2D (19). Poor glycemic control in individuals with T2D results in severe complications, such as renal failure, blindness, neuropathy, and CV disorders (20).

**Figure 1:**
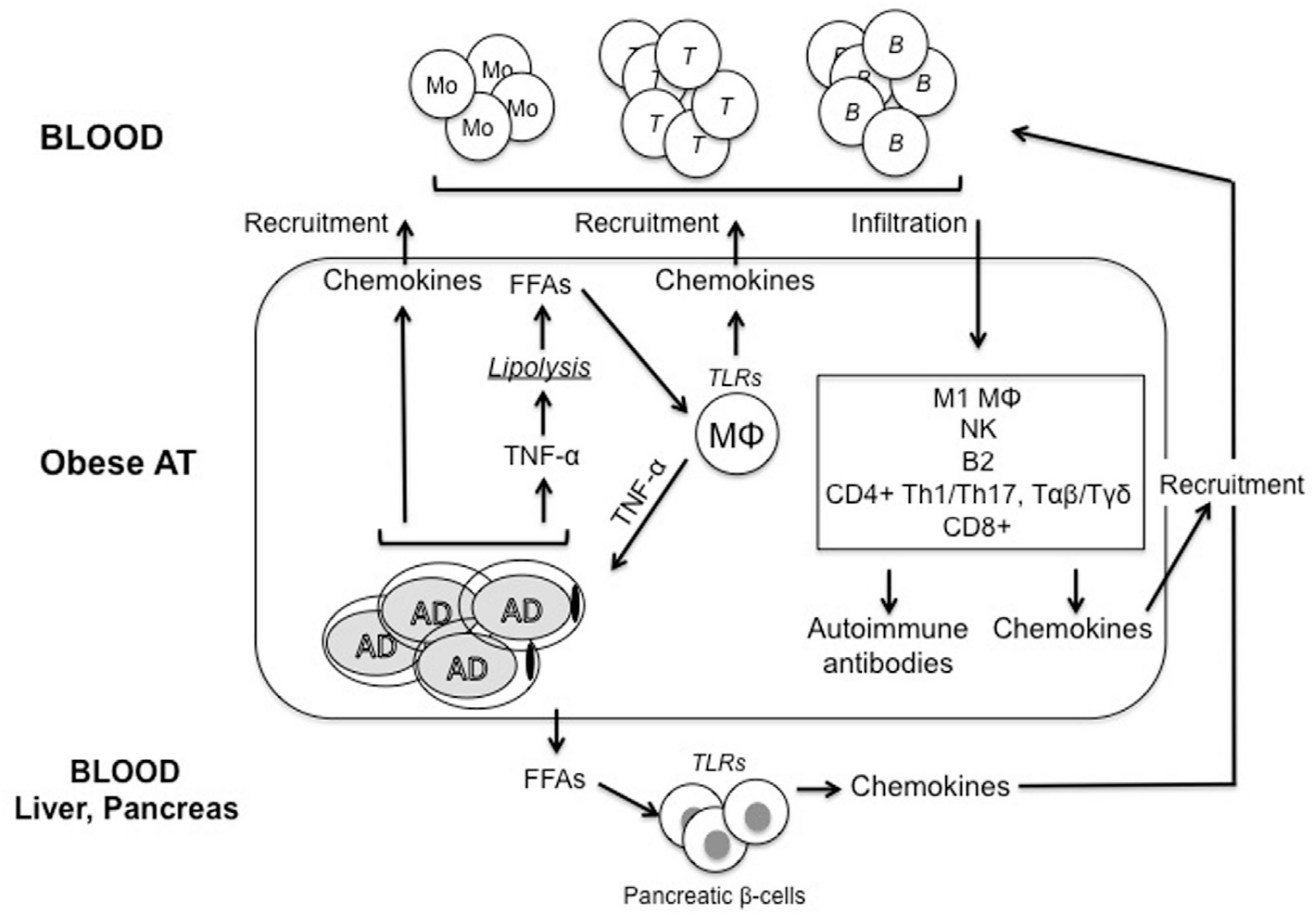
Model for regulation of inflammatory pathways in the obese adipose tissue (AT). Adipocytes (AD) in the obese AT are highly inflammatory and secrete several pro-inflammatory cytokines and chemokines, which recruit immune cells, thus contributing to the establishment and maintenance of local and systemic inflammation. Among these inflammatory mediators, tumor necrosis factor (TNF)-α released by both AD and immune cells induces lipolysis and release of free fatty acids (FFAs), which activate tissue-resident macrophages (MΦ) to release cytokines and chemokines. FFAs are also released in blood and cause both insulin resistance and inflammation in major insulin target tissues. Immune cells recruited to the obese AT differentiate into inflammatory subsets and secrete additional pro-inflammatory mediators. We hypothesize that these cells would generate suboptimal immune responses in obese individuals by circulating to peripheral lymphoid organs. Pathogenic antibodies may be secreted by B cells in the AT. These antibodies may form immune complexes with “self”-antigens, which in turn activate complement and Fc receptors on immune cells, leading to enhanced local inflammation, remodeling of the AT, impairment of adipocyte function and nutrient metabolism, and exacerbation of obesity-associated conditions. These antibodies can also exert additional detrimental effects both locally and systemically targeting distinct clusters of self proteins. One mechanisms for the release of “self”-antigens in the obese AT is the decreased supply of oxygen, resulting in areas of hypoxia, which leads to further release of pro-inflammatory cytokines, as well as to the release of “self”-antigens, such as intracellular proteins, cell-free DNA, and lipids.

It is not completely clear how obesity causes the development of IR. Although many molecular mechanisms have been proposed, including ER stress, oxidative stress, dysregulation of lipid homeostasis, mitochondrial dysfunction, hypoxia, and impairment of the insulin signaling pathway in insulin-responsive cells, there is evidence that obesity-induced inflammation may be a key factor for IR (21). Figure 1 summarizes the main pathways leading to inflammation in the obese AT.

### Production of Pro-inflammatory Mediators in the Obese AT

High levels of TNF-α in the AT are associated with chronically elevated basal lipolysis, the process of hydrolysis of tryglycerides to release FFAs and lipids (22). These provide chronic stimulation to macrophages leading to FFA-induced TNF-α production, causing IR. It has been proposed that adipocyte-derived TNF-α contributes to elevated levels of FFAs in the blood of obese individuals (22), and neutralization of TNF-α *in vivo* in obese mice decreases circulating levels of FFAs (23). TNF-α has also been shown to reduce the expression of proteins stabilizing lipid droplet (perilipins) (24), leading to ectopic lipid deposition in insulin-sensitive tissues. Lipids and lipid-derived molecules have direct effects on insulin-sensitive tissues and induce IR (25).

Other major pro-inflammatory cytokines released by the obese AT are IFN-γ secreted by CD8^+^ T cells (26) and NK cells (27) and IL-17 secreted by CD4^+^ T cells (28).

### Hypoxia and Release of “Self” Antigens in the Obese AT

During the development of obesity, the supply of oxygen to the expanding AT becomes inadequate, resulting in areas of hypoxia (29, 30). This phenomenon of poorly oxygenated AT not only activates the transcription factor hypoxia-inducible factor-1α (HIF-1α) and further release of pro-inflammatory cytokines (31) but also induces cell death and release of “self” antigens, which stimulate class switch and the production of IgG pathogenic antibodies. Hypoxia in the AT has been the only mechanism suggested so far for the release of “self” antigens in the obese AT.

### Immune Cell Infiltration in the Obese AT

Data from obese mice and humans have indicated that the hypertrophied AT becomes heavily infiltrated by a variety of immune cells displaying a pro-inflammatory phenotype, characterized by secretion of SASP markers (32), and their numbers inversely correlate with insulin sensitivity. Cells with an anti-inflammatory phenotype have also been reported in the obese AT, but these cells are present at low frequencies. These are B1 B cells producing IL-10 (15, 33) and innate lymphoid cells type 2, which produce large amounts of Th2 cytokines such as IL-4, IL-5, and IL-13 (34). Tregs have also been reported but only in the lean AT (35).

Macrophage infiltration within the AT has been considered a major driver of inflammation, due to the secretion of pro-inflammatory cytokines and chemokines involved in the recruitment of immune cells to the AT. However, adipocytes also secrete pro-inflammatory mediators (cytokines, chemokines, and adipokines) and in larger amounts compared with immune cells (36). Therefore, with obesity, a crosstalk between adipocytes and the immune cells infiltrating the AT contributes to the establishment of chronic inflammation, a prerequisite for IR. Macrophages in the AT are almost exclusively M1, they depend on glycolysis for their inflammatory function, and their stimulation in the AT induces glucose transporter expression and glucose intake and utilization (37). Hypoxia (via HIF-1α) potentiates glycolysis and stabilizes the inflammatory phenotype (38). In M1 macrophages, the inflammasome NLRP3 activates caspase 1 and the secretion of IL-1β (39), which is directly toxic to pancreatic β-cells and induces IR (40). Increased inflammasome activity has been reported in monocyte-derived macrophages from T2D patients (41).

T cells in the AT are Th1 CD4^+^ and IFN-γ-producing CD8^+^ T cells (26). These promote secretion of pro-inflammatory cytokines from M1 macrophages leading to both local and systemic IR (42). Similar to macrophages, T cell subset skewing in the AT occurs through modulation of substrate metabolism regulated by hormones (leptin) and intracellular nutrient sensing kinases, such as AMPK/mTOR (43). Th1 CD4^+^ T cells express high levels of membrane glucose transporters and are highly glycolytic (44), a trait supporting inflammatory responses.

Interferon-γ, the signature Th1 cytokine, induces macrophages and T cells to secrete chemokines, which recruit immune cells to the obese AT (45, 46). Moreover, IFN-γ facilitates the M2 to M1 polarization (47) and decreases insulin receptor signaling by reducing the expression of insulin receptors and glucose transporters (48). IFN-γ production is regulated by T-bet, a T-box family transcription factor first identified as a transcriptional inducer of IFN-γ in CD4^+^ T cells (49). T-bet plays a critical role in the development of IR in animal models of obesity, and T-bet-deficient mice fed a high-fat diet are refractory to the induction of IR (50). These mice show improved insulin sensitivity and glucose tolerance, reduced numbers of immune cells in the AT (CD4^+^/CD8^+^ T cells, NK cells, and macrophages), and reduced production of pro-inflammatory cytokines per gram of fat (IFN-γ, TNF-α, IL-1β, and IL-6).

Obese and T2D patients have alterations in the composition of their microbiome, with reduced proportions of Bacteroidetes (beneficial bacteria) in obese versus lean individuals (51). Moreover, it has been reported that the gut microflora regulates the development of obesity in animal models (52). T-bet regulates mucosal T cell activation (53), and T-bet deficiency alters the composition of microflora (54). T-bet deficiency may also alter the microbiome in individuals with obesity leading to the inflammatory and metabolic processes that regulate T2D.

B cells also accumulate in the obese AT (15, 55, 56). B cell recruitment can initiate T cell-induced M1 polarization and IR. Obesity and hyperglycemia have direct influence on antibody production, and IgG secretion from inflamed VAT modulate the function of resident macrophages. It has been reported that B cells in AT are induced to produce pathogenic IgG autoantibodies, due to the dysregulated expression of autoantigens by hypoxic adipocytes. B cells also support the activation of inflammatory T cells, which are the main pathogenic drivers in systemic inflammation and IR.

Recently, a new lymphoid tissue called fat-associated lymphoid clusters (FALCs) has been identified in the mesenteric AT of mice and humans. FALCs are rapidly induced after inflammatory stimuli and support B cell proliferation and differentiation regulating antibody production within the AT (57).

## Obesity and Rheumatoid Arthritis (RA): Evidences and Mechanistic Links

RA is a debilitating chronic autoimmune disease that causes synovial inflammation and destruction of joints including the cartilage and the adjacent bone. It generally occurs between the fourth and sixth decades of life and affects more women than men. It is characterized by joint stiffness, pain, and swelling and is accompanied by extraarticular manifestations and systemic inflammation. RA has been associated with muscle wasting and cachexia due to uncontrolled inflammation driven by TNF-α, which fuels hypercatabolism (58). However, BMI rarely falls below normal because loss of lean tissue is compensated by increased AT, and this characterizes rheumatoid cachexia, also called “cachectic obesity” (59, 60). It has been observed that in RA patients, despite adequate nutrient intake, but inflammatory cytokine dominance and reduced activity due to pain, joint deformity, and decreased muscle strength (61), cachexia appears to be similar to that occurring in aged subjects with disability. Abnormal body composition in RA can be defined as a sarcopenic obesity (62) with characteristic changes that in the elderly contribute to frailty (63, 64). Moreover, the percentage of obese RA patients has increased (65), and the impact of obesity on RA has become a relevant issue not much for the negligible risk of developing RA (66), but for its negative effects on disease activity, response to therapy, and CV risk. Obese RA patients are indeed less likely to achieve sustained remission in response to therapy with conventional chemical (4) or biologic (TNF-α inhibitors) agents (67). Despite opposite results with some treatments (68), obesity decreases the rate of remission in RA and negatively affects disease activity (69) and patient-reported outcomes during therapy (70).

### The “Obesity Paradox” in RA

Obesity represents an important link with comorbidities such as metabolic syndrome (71) and CV diseases (72); however, in some studies, increased BMI had the opposite effect of reduced mortality (70, 73), which has been described as the “obesity paradox” (74). Moreover, in overweight RA patients, progression of bone destruction was reduced (75, 76), the number of swollen joints is not increased, and better quality of life has been reported (77). Weight loss and cachexia represent major determinants for a greater risk of death (78) and worse quality of life (77), thus strengthening the paradoxical observation of lower mortality in obese patients. However, follow-up studies have demonstrated that in RA patients with a history of obesity reduced BMI is strongly associated with death. Therefore, the “obesity paradox” does not entail a biologically protective role of obesity (73), raising the question whether the use of BMI is a valid tool for assessing obesity in RA (65).

High BMI contributes to disease activity in RA by affecting both biomechanics and the metabolic status, and obese RA patients show worse subjective assessment of symptoms (79). Hyperglycemia, as a part of the metabolic syndrome, is more common in early RA (80), whereas active RA shows decreased lipid levels (81) despite an increased risk of CV events, due to the lipid-lowering effect of systemic inflammation (82). An increase in VAT, e.g., the epicardial fat (83), and the more abundant macrophage infiltrate are associated with systemic inflammation, metabolic syndrome, and IR (84). Anti-TNF-α therapy improves insulin sensitivity in RA patients who are resistant, but despite controlling inflammation, it does not achieve the same extent of improvement in obese RA patients (85). In addition, even when therapy succeeds in the control of disease activity, it fails to restore the altered body composition and improve physical function (86). Adipokines (leptin, adiponectin, visfatin, resistin, and chemerin) have been postulated to be the mediators linking AT and RA activity (87). Adipokine imbalance may underlie the higher degree of inflammation (88), the levels of autoantibodies (leptin and adiponectin differentially regulate the generation of Treg cells, which are abundant in normal VAT), and also the lower amount of bone resorption observed in obese patients (89). On another level, the association of RA with both the metabolic syndrome and atherosclerosis is probably also mediated by VAT through altered secretion of adipokines. Therefore, adipokines contribute decisively to the systemic inflammation underlying RA, which represents an independent risk factor for CV diseases.

Since weight reduction may have possible contraindications (lower BMI being associated with accelerated mortality in RA), and the assessment of the inflammatory milieu of VAT in RA patients is still incomplete, much research has been devoted to uncovering the metabolic changes occurring in the development and chronicization of RA. This field has been recently reviewed (90) and can be summarized in the two distinct stages of early and chronic RA. In the first stage, there is a high metabolic demand in all cell types involved, due to proliferative signaling, angiogenesis, cellular de-differentiation, and unbalanced bone turnover. However, in RA T cells, at variance with other types of inflammatory metabolic changes, the glycolytic pathway is reduced in favor of the pentose phosphate shunt (91), reduced ROS generation, and decreased AMPK function. In early stages, AMPK activation (e.g., by Metformin) may be an attractive target because its activity is decreased in several tissues of obese or IR patients. In the late (erosive) stage of RA, the inflamed joint is a hypermetabolic lesion (90), T cells undergo a metabolic switch to aerobic glycolysis due to hypoxic conditions, and mitochondrial dysfunction with increased lactate production causes acidification of the synovia. The reprogramming of T cells accounts for pro-inflammatory Th1/Th17 phenotypes and premature T cell aging (92). Several aspects of immunosenescence have been found to be relevant in RA pathogenesis (93–96), and rejuvenation of the immune system has been proposed as therapy, including mTOR inhibitors (97, 98).

### Role of B Cells in RA Pathogenesis

The key role played by autoreactive B cells is highlighted by the presence of diagnostic autoantibodies, and rheumatoid factor (RF) (99) and anticyclic-citrullinated peptide antibodies (ACPAs) (100) are well-established indicators of disease and disease severity and may precede the onset of disease. The role of B cells in RA pathogenesis in the context of overweight/obesity has not been addressed yet and deserves thorough attention. A primary defect in early B cell tolerance has been detected since the majority of naive B lymphocytes express polyreactive autoantibodies, including RF and ACPA. These B cells are resistant to Fas-induced apoptosis and therefore not suppressed by Treg (101). However, B cells are involved in RA by other mechanisms, in a bidirectional support of helper T lymphocytes, as self-antigen-presenting cells, with the release of inflammatory mediators, and with the promotion of lymphoid neogenesis (which is prominent in RA synovitis). RF^+^ B cells are able to take up IgG-containing immune complexes and present antigen to T cells, thus activating a reciprocally reinforced response (102).

The phenotype of B lymphocytes in RA has been extensively studied in peripheral blood and in synovial tissue, with some discordant data owing to examination of different stages of the disease. The general consensus is the increased presence of memory (CD27^+^) B cells with an activated (CD95^+^, CD21^low^) phenotype both in peripheral blood and in the synovial compartment (103). These cells increased even more significantly after B cell depletion therapy (BCDT) with rituximab. There is also agreement on the fact that response to BCDT relies on elimination of memory B cells, and their repopulation, along with transitional B lymphocytes, may predict relapse (103). The role of homeostatic lymphoproliferation of both memory B cells and the extent of BCDT in bone marrow and synovial tissue represent critical points still to be elucidated. Since it has been observed that CD4^+^ T cell activation decrease after BCDT (104), changes in not only B cell subsets but also T cell subsets may underlie the response of RA patients to therapy. The Treg compartment is less affected by RA treatments (105), but the presence of Breg lymphocytes (decreased in untreated RA) seem to play a role in balancing immune abnormalities and predict the treatment outcome (106). Cytokine production by B cell subsets is also relevant to RA pathogenesis and disease activity [reviewed in Ref. (107)], with inflammatory cytokines predominating in untreated severe RA, as activated memory B cells preferentially secrete TNF-α, whereas BCDT induced a shift to subpopulations producing IL-10. The recent identification of a subset of B cells able to produce large amounts of RANKL (108) provides a mechanistic link between activated memory B cells and bone resorption through induction of osteoclastogenesis. It is relevant to mention that ACPAs are associated with more joint and bone damage and that therapy does not eliminate ACPA-producing autoreactive B cells in the synovial tissue. The central role of B lymphocytes in RA pathogenesis and in tissue damage makes these cells and their products attractive targets for treatment; however, there is still uncertainty about the beneficial or even protective effects of B cell subsets.

## Osteoarthritis (OA), Aging, and Obesity

In the elderly, arthritis is frequently associated with other diseases with multiple aging or degenerative features (109). OA and RA share common features in elderly patients and significantly contribute to disability (110). OA is usually differentiated from RA by age at diagnosis, duration of morning stiffness, pattern of joint involvement, and radiographic findings. Distinguishing between the diseases can be challenging, but in the >60 years of age group, OA is by far more common. Despite the fact that OA directly correlates with age, the real cause of this association is not clear, and OA development can be separated into aging-dependent and aging-independent processes (111–114). Both increased production of matrix metalloproteinases and cytokines, reduced levels of collagen type II synthesis, and increased production of ROS induce age-related changes in chondrocytes (114). These changes alter cartilage function, and sarcopenia further leads to decreased joint stability (115). Cellular senescence, impaired regeneration, and repair are recognized factors contributing to cartilage damage with aging (115, 116).

In patients younger than 60 years of age with symptomatic OA, joint pain and disabilities are less recognized as inevitable consequences of growing old, compared to OA patients older than 70 years (117). Several factors contribute to the development of OA: acute injury (including fracture), excessive mechanical overloading (113, 118), diabetes, and chronic tobacco smoking, all playing a role in the amplification of senescence-inducing stresses (118–121). These factors develop before symptoms appearance and may cause early onset of OA; multimorbidity including OA and obesity can be seen at an adult age (122). The prevalence of arthritis is increasing, with 29.3% ever reported doctor-diagnosed arthritis in individuals aged 45–64 years versus 49.6% in individuals aged 65 or older in the United States (123). However, obesity prevalence did not change significantly over time among middle-aged and younger adults with doctor-diagnosed arthritis (124) despite increasing significantly over time among older adults with RA and remaining also higher when compared with adults without RA. Obesity impacts progression of OA and has a negative influence on outcomes (125). Exercise and loss of at least 10% of body weight can effectively lead to improvement in symptoms, pain relief, and physical function. Physical activity may reactivate a regenerative process by mobilizing stem cells and increase proteoglycan production, restoring the cartilage structure (113, 115, 126).

## Cognitive Impairment, Dementia, and Relationship to Age-Associated Diseases and Obesity

Our summary of conditions where inflammation, obesity, and aging converge in defining particular features and outcomes of disease must also briefly mention the dementias, whose prevalence has been reported to be declining among older US adults between 2000 and 2012 (127). However, dementia rates are growing at an alarming proportion in most regions of the world and are related to population aging (128). Prevalence varies in countries with different mean population ages. However, differences persist after adjusting for age (129). The decline in the United States occurred in those older than 65 years and was related to increased number of years in education despite the age- and sex-adjusted increase in the prevalence of hypertension, diabetes, and obesity in the same years. There is a long unresolved debate on the prodromal phase of the neurodegenerative disorders with inflammatory features, such as Alzheimer’s dementia, but it is undisputed that prevalence of dementias of all types increase with old age, from about 2–3% among those aged 70–75 years to 20–25% among those aged 85 years or more (130). The known risk factors include obesity, depression, diabetes, decreased physical activity, hypertension, smoking, hypercholesterolemia, coronary heart disease, and alcohol use; and assessment of these provide an estimate of the risk of developing dementia (131) despite the fact that in the oldest-old (80–97 years old), these factors did not increase the risk for dementia, so that age plays a major role.

Taken together for the two most frequent types of dementia (Alzheimer’s and Vascular) (129), vascular risk factors such as dietary fat intake, high cholesterol, obesity, T2D, and hypertension have emerged as the most important determinants (132). Vascular risk is seldom isolated and is accompanied by alterations in hormonal metabolism. Overweight/obesity, due to excess AT, increase the CV risk and also for late-onset dementia. This is exemplified in the prodromal phases of dementia, as vascular and metabolic parameters decline in direct relation to cognitive impairment and in a way which seems to differ from that occurring in “normal” aging. With regard to obesity, its presence at midlife is associated with an increased risk of dementia and Alzheimer’s later in life (133), and in particular central obesity in midlife increases the risk of dementia independent of diabetes and CV comorbidities (134). The risk is reversed when late-life BMI is considered: underweight persons had an increased risk of dementia, whereas being overweight was not associated and being obese reduced the risk of dementia compared with normal BMI. This has been dubbed as an “obesity paradox” also in this case (135). A recent systematic review and meta-analysis suggests a positive association between obesity in mid-life and later dementia but the opposite in late life (136). A successive study confirmed the association of mid-life obesity and dementia, but that of being underweight and dementia remained controversial (137). It is difficult to draw a clear distinction between visceral adiposity and total body fat in most studies, and this is reflected on the paucity of mechanistic hypotheses supported by experimental data. The attention has been focused on the role of several adipokines and mainly the two major hormones produced by the AT, leptin and adiponectin, that interact directly with the brain (138). They have the capability to cross the blood–brain barrier and influence dementia processes within the brain (139), but evidence for a direct role is missing. Another postulated link is through altered gut microbial flora, which may participate in the development of obesity, T2D, and subsequent initiation of AD (140). Also lacking is the evidence that weight reduction in mid-life may produce beneficial effects on dementia development. However, in older adults, regular exercise provides numerous health benefits that include improvements in blood pressure, coronary artery disease, diabetes, lipid profile, OA, osteoporosis, mood, neurocognitive function, and overall morbidity so that studies in this area should be encouraged.

## Concluding Remarks

Immunity and metabolism are highly integrated factors in aging and age-related diseases. This is an expanding field of investigation. Obesity and related complications are a major global epidemic. Scientific research must be a crucial part of the solution to understand all implications of obesity, but this research is still in its initial phase. The investigation of the mechanisms whereby inflammation and immune activation disrupt a functional immune response adds novel insights to the understanding of the relationship between inflammation and long-term metabolic disease outcome and opens new ways for effective therapeutic interventions.

## Author Contributions

All authors were involved in writing the article and had final approval of the submitted version.

## Conflict of Interest Statement

The research was conducted in the absence of any commercial or financial relationships that could be construed as a potential conflict of interest.
